# Supplementary material for: Reflecting glory or deflecting stigma? The interplay between status and social proximity in peer evaluations
Source: PLoS One. 2020 Sep 25;15(9):e0238651. doi: 10.1371/journal.pone.0238651 (PMC7518619; doi:10.1371/journal.pone.0238651)
Supplement: S1 Table — (DOCX) [file pone.0238651.s001.docx]

**S1 Table. Results for Study 4**

|  | Status | | No Status | | | |  |
| --- | --- | --- | --- | --- | --- | --- | --- |
|  | Social  Ties  (A) | No  Ties  (B) | | Social  Ties  (C) | No  Ties  (D) | Interaction  F (1, 410) | Simple Effects |
| Award Propensity  M | 4.2 | 4.52 | | 4.55 | 4.18 | 8.27  (*p*=.004) | B > D (*p*=.043)  A < C (*p*=.042) |
| N | 105 | 104 | | 98 | 107 |  |  |
|  | | | | | | | |
