# Supplementary figures and images for: Reflecting glory or deflecting stigma? The interplay between status and social proximity in peer evaluations
Source: PLoS One. 2020 Sep 25;15(9):e0238651. doi: 10.1371/journal.pone.0238651 (PMC7518619; doi:10.1371/journal.pone.0238651)

**S1 Figure. Study 4: The effect of *Status* and *Social Ties* on *Award Propensity***

**
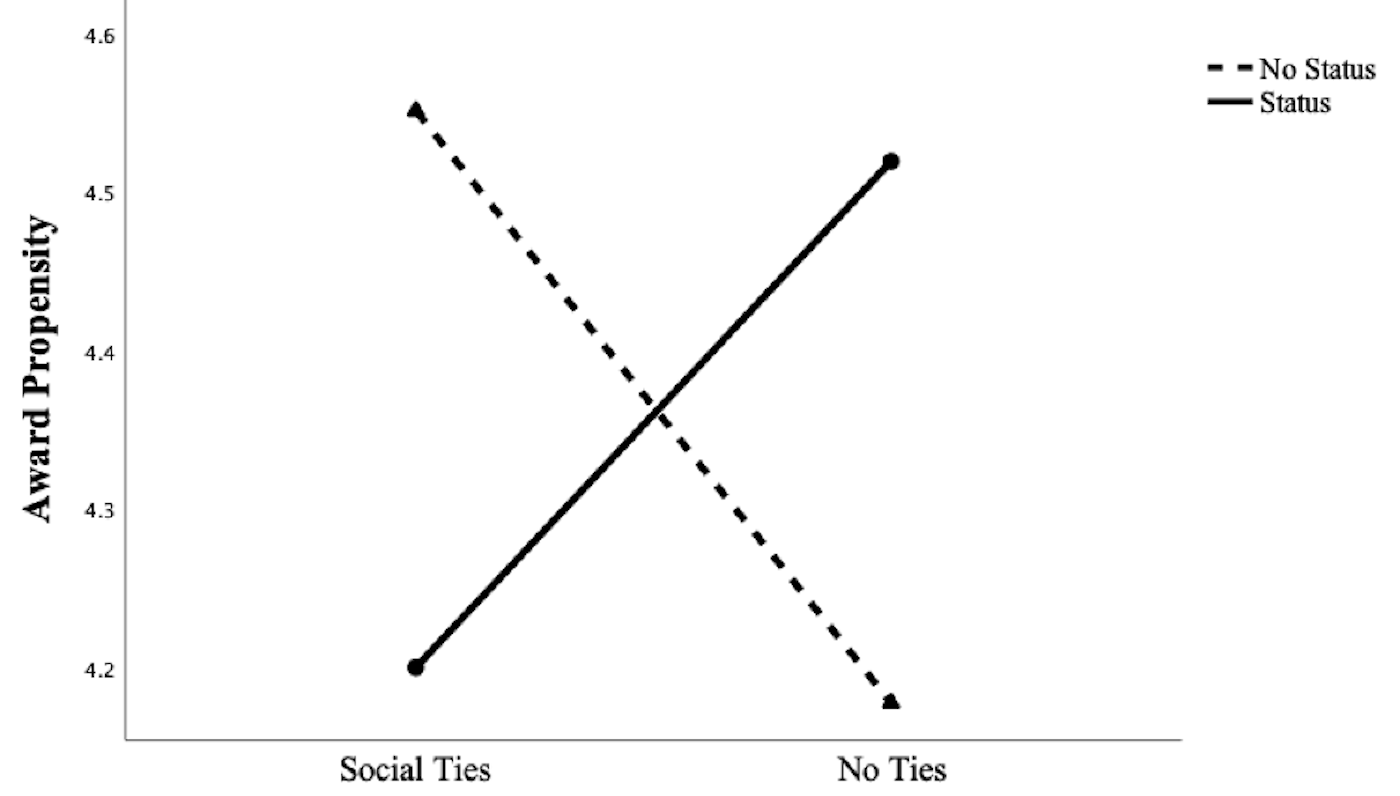
**

Supplement: S1 Fig — (DOCX) [file pone.0238651.s002.docx]

**S2 Figure. Study 4: The effect of *Status* and *Social Ties* on *Award Propensity***

**
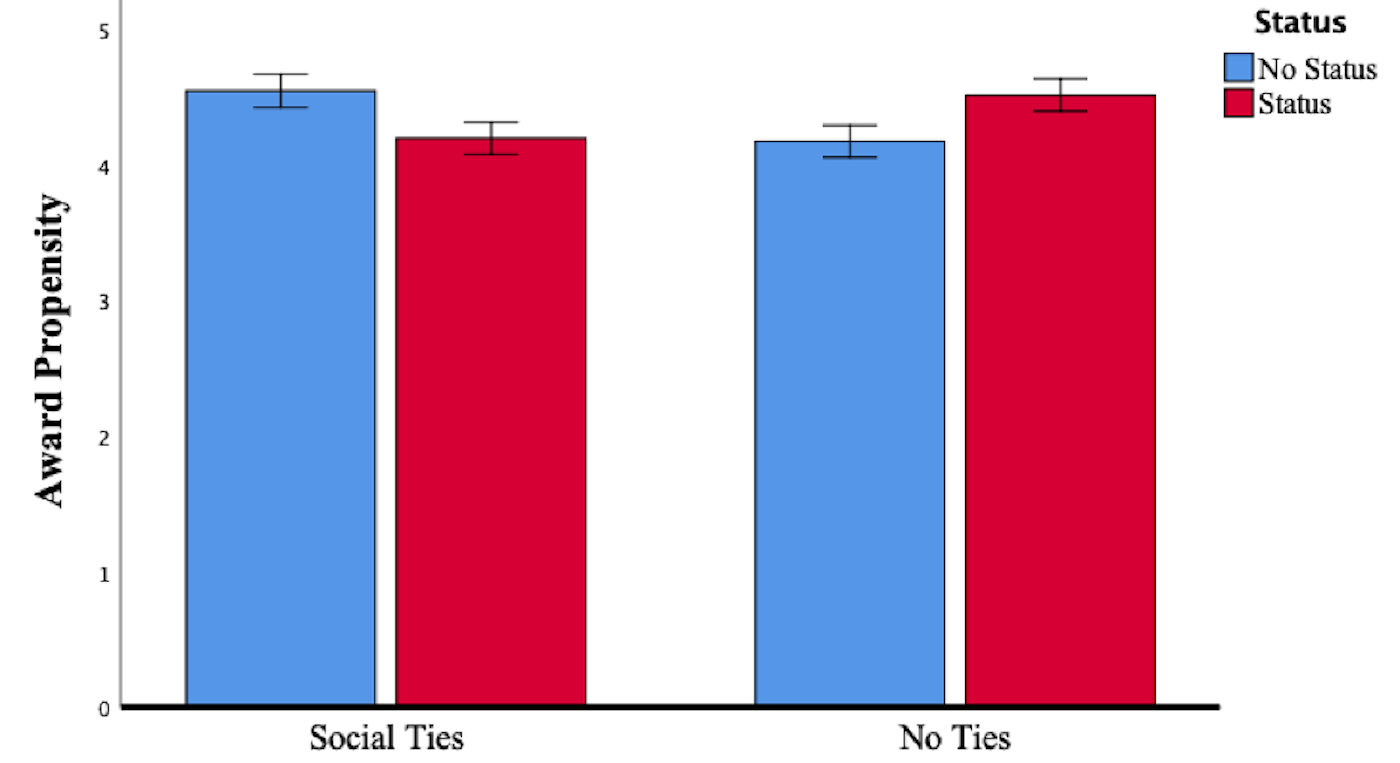
**

*Note*: Error bars are ± 1 SE

Supplement: S2 Fig — (DOCX) [file pone.0238651.s003.docx]
