# Supplementary material for: Reflecting glory or deflecting stigma? The interplay between status and social proximity in peer evaluations
Source: PLoS One. 2020 Sep 25;15(9):e0238651. doi: 10.1371/journal.pone.0238651 (PMC7518619; doi:10.1371/journal.pone.0238651)
Supplement: S1 Appendix — (DOCX) [file pone.0238651.s004.docx]

**S1 APPENDIX**

**STUDY 4**

We replicated Study 2 by conducting another online experiment with a different scenario – in which we asked participants to imagine working for an advertising company – and a different manipulation of status. Like in Study 2, all participants were asked to evaluate the same commercial and informed that their evaluations were in the public domain (i.e., the jury selected the winners collectively). We used the same dependent variable – *award propensity* – employed in the prior studies.

**Method**

***Participants****.* Five hundred and one participants were recruited online using Amazon’s Mechanical Turk and received $.80 for completing the study. Like in Study 2, we prescreened potential participants who were US residents and had at least a 95% approval rating on MTurk. Since we asked them to imagine working for an advertising company, we used the MTurk qualification filter to recruit only full-time employed participants. To detect careless responses, we employed the same two attention checks used in Study 3 (i.e., a question regarding the commercial and an IMC to leave blank the question), and excluded the participants who did not answer correctly. To be consisted with our prior experiments, we asked them to evaluate the same commercial and included in the analysis only those who watched the commercial for more than 50 seconds and less than 122 seconds. The final sample consisted of 416 participants (52.8% female, *Mage*=40.36 years, 83.5% Caucasian).

***Material and Procedure***. We randomly assigned the participants to one of the four conditions in a 2 (status: status vs. no-status) x 2 (social ties: direct ties vs. no-direct ties) between-subjects experiment. We instructed the participants to imagine that they work in a company in the advertising industry and that this year they qualified to become jury members of a digital advertising competition as they participated in it and won in the past. Consistently with Study 2, we asked them to assign an award to a commercial after evaluating its aesthetic beauty and animation features, and also informed them that “The jury selects the winner collectively thereby disclosing the vote cast by each jury member.” Specifically, the evaluative setting was described as follow:

**Advertising Digital Competition**

“Imagine that you work in a company in the advertising industry.

Each year there is a competition in Digital Advertising and everyone in the industry can participate in the competition by submitting a commercial. Each commercial is judged and has the opportunity to win an award.

Since you participated and won the competition in the past, this year you qualified to become a **jury member** of the Digital Advertising competition. As a jury member, you have to assign an award to a commercial after evaluating its **aesthetic beauty** and **animation features**.”

Then, the participants received more information about the commercial’s creators (*authors* in the vignettes) whose description was varied according to the creators’ *status* and *social ties* with the experimental participants. Specifically, we manipulated status by describing the creators of the commercials as ‘famous professionals’ in the status condition, and ‘not very famous professionals’ in the no-status condition. To manipulate social ties, we applied the same manipulation of Study 2. In sum, the participants in the status and social ties condition read the description below (if assigned to the no-status and no-direct ties conditions, the participants read the text in italics):

“In addition to the video, the organizers provide you with some information about the authors of the commercial. Looking at this information, you realized that all the authors of the commercial are **famous** (***not very famous***) **professionals** in advertising, and that you **know** some (***don’t know any***) of them because you **collaborated with** them (***never collaborated with them***) on commercials in the past.”

Finally, the participants watched and evaluated the same commercial on the financial service used in our prior experiments.

***Award Propensity***. The same question from the prior experimental studies was employed to measure the propensity to award the commercial.

***Manipulation Checks***. The same questions from Study 2 was used for the manipulation check of status and social ties.

**Results and Discussion**

*Pre-analysis*. Like in the previous experiments, we identified two outliers for our dependent variable based on the Z-scores threshold of 2.5 SD [62, 53]. These subjects were removed from all subsequent analyses.

*Manipulation checks*. A 2 x 2 ANOVA on the rating of the creators’ prestige confirmed the appropriateness of the status manipulation: the participants in the *status* condition (M = 4.89, *SD*= 1.25) rated the commercial’s creators as more prestigious than the participants in the *no-status* condition (M= 3.01, *SD*= 1.27; F (1, 410) = 229.06, *p*<.001). We found no other significant effects in the results. Thus, the manipulations of status worked well.

The social tie manipulation was also confirmed in a 2 x 2 ANOVA on the rating of the creators’ familiarity: the participants in the direct ties condition (M= 3.13, *SD* = 1.7) considered the commercial’s creators as more familiar than the participants in the no-direct ties condition (M= 1.56, *SD*= .98; F (1, 410) = 135.35, *p*<.001). We found also a marginally significant interaction effect (F (1, 410) = 3.36, *p*=.068), while the main effect for status was not significant. To ensure our experimental manipulation was not confounded, we compared the effect sizes by following Perdue and Summers’ [60] procedure. The effect size of the social tie manipulation (η^2^_social ties_=.248) was 31 times greater than the effect size of the interaction effect (η^2^_intercation_=.008), suggesting that our social tie manipulation worked as expected.

*Award Propensity.* A 2 x 2 ANOVA on award propensity revealed a significant two-way interaction (F (1,410) = 8.27, *p*<.01), while the main effects of status and social ties were not significant. Consistently with our expectation, simple effects tests showed that the participants with direct ties to the commercial’s creators were less willing to assign the commercial an award when creators with status (M = 4.20, *SD* = 1.3) rather than no-status (M = 4.55, *SD* = 1.14; F (1,410) = 4.17, *p*<.05) were involved. In contrast, the participants with no direct ties to the commercial’s creators were more willing to assign the commercial an award if status (M = 4.52, *SD* = 1.12) rather than no-status (M = 4.18, *SD* = 1.32; F (1,410) = 4.11, *p*<.05) creators were involved. S1 Fig graphs the lines, S2 Fig reports the bar charts, and S1 Table reports the results.

In sum, with this additional experiment, we replicated the experimental results of Study 2 by using a different manipulation of status and, again, provided evidence of the negative interaction of status and social ties found in our field study.
