# Supplementary material for: Reflecting glory or deflecting stigma? The interplay between status and social proximity in peer evaluations
Source: PLoS One. 2020 Sep 25;15(9):e0238651. doi: 10.1371/journal.pone.0238651 (PMC7518619; doi:10.1371/journal.pone.0238651)
Supplement: Attachment — Submitted filename: Response to Reviewers.docx [file pone.0238651.s006.docx]

*PONE-D-19-12690
The Interplay between Status and Social Proximity in Peer Evaluation:
A Socio-Cognitive Perspective on Reward Allocation
PLOS ONE

Dear Prof. Cattani,

Thank you for submitting your manuscript to PLOS ONE. After careful consideration, we feel that it has merit but does not fully meet PLOS ONE’s publication criteria as it currently stands. Therefore, we invite you to submit a revised version of the manuscript that addresses the points raised during the review process.*

**We appreciate the time and thought you have put into your evaluation of our manuscript. Your comments highlighted areas for improvement and, below, we explain how we have responded to your comments. For the sake of convenience, we present your comments (in italics) first and then our response.**

*Please find below the reviewers' comments. We would appreciate receiving your revised manuscript by Oct 24 2019 11:59PM. When you are ready to submit your revision, log on to* [*https://www.editorialmanager.com/pone/*](https://www.editorialmanager.com/pone/) *and select the 'Submissions Needing Revision' folder to locate your manuscript file. If you would like to make changes to your financial disclosure, please include your updated statement in your cover letter.

To enhance the reproducibility of your results, we recommend that if applicable you deposit your laboratory protocols in protocols.io, where a protocol can be assigned its own identifier (DOI) such that it can be cited independently in the future. For instructions see:* [*http://journals.plos.org/plosone/s/submission-guidelines#loc-laboratory-protocols*](http://journals.plos.org/plosone/s/submission-guidelines#loc-laboratory-protocols) *Please include the following items when submitting your revised manuscript:*

- *A rebuttal letter that responds to each point raised by the academic editor and reviewer(s). This letter should be uploaded as separate file and labeled 'Response to Reviewers'.*
- *A marked-up copy of your manuscript that highlights changes made to the original version. This file should be uploaded as separate file and labeled 'Revised Manuscript with Track Changes'.*
- *An unmarked version of your revised paper without tracked changes. This file should be uploaded as separate file and labeled 'Manuscript'.*

*Please note while forming your response, if your article is accepted, you may have the opportunity to make the peer review history publicly available. The record will include editor decision letters (with reviews) and your responses to reviewer comments. If eligible, we will contact you to opt in or out.*

*We look forward to receiving your revised manuscript.

Kind regards,

Valerio Capraro
Academic Editor
PLOS ONE*

**We thank you for your consideration of our work. As we explain in this Response to the Reviewers, we tried our best to carefully address all of their comments and revise the manuscript accordingly. We hope you will enjoy this revised version.**

*Journal Requirements:*

*When submitting your revision, we need you to address these additional requirements. Please ensure that your manuscript meets PLOS ONE's style requirements, including those for file naming. The PLOS ONE style templates can be found at*

[*http://www.journals.plos.org/plosone/s/file?id=wjVg/PLOSOne_formatting_sample_main_body.pdf*](http://www.journals.plos.org/plosone/s/file?id=wjVg/PLOSOne_formatting_sample_main_body.pdf) *and* [*http://www.journals.plos.org/plosone/s/file?id=ba62/PLOSOne_formatting_sample_title_authors_affiliations.pdf*](http://www.journals.plos.org/plosone/s/file?id=ba62/PLOSOne_formatting_sample_title_authors_affiliations.pdf)

**The manuscript now meets PLOS ONE’s style requirements, including those for file naming.**

*1. Please provide additional details regarding participant consent for study 1 (interviews with a panel of field insiders and identified individuals in “The Silver Tag” data set. In the ethics statement in the Methods and online submission information, please ensure that you have specified (1) whether consent was informed and (2) what type you obtained (for instance, written or verbal). If your study included minors, state whether you obtained consent from parents or guardians. If the need for consent was waived by the ethics committee, please include this information in the manuscript.*

**We obtained oral informed consent from all interviewees prior to conducting our interviews. All interviewees gave interviews under the condition of anonymous treatment. The advertising agencies in which the interviewees worked knew about the study, the purpose of the study, and provided us with access to the interview subjects in their respective firms.**

*2. We note that you have stated that you will provide repository information for your data at acceptance. Should your manuscript be accepted for publication, we will hold it until you provide the relevant accession numbers or DOIs necessary to access your data. If you wish to make changes to your Data Availability statement, please describe these changes in your cover letter and we will update your Data Availability statement to reflect the information you provide.*

**We are happy to make the data available should the paper be accepted for publication.**

*3. Please ensure that you include a title page within your main document. You should list all authors and all affiliations as per our author instructions and clearly indicate the corresponding author.*

**We have included a title page within our main document that lists all authors and all affiliations.**

*4. Please amend the file type of your supporting information files to 'other'*

**We have amended the file type of our supporting information files to “other.”**

Additional Editor Comments (if provided):

*I have now collected two reviews from two experts in the field. They both think that the manuscript has merit, but raise several concerns that somewhat diminish their enthusiasm. As a consequence, one of the reviewer recommends major revision, while the other one recommends rejection (although s/he acknowledges that a revision of the paper might be successful. Therefore, I have opted for offering you the chance to revise your work, following the reviewers' comments. Needless to say that all comments must be addressed. I am looking forward for the revision.*

*[Note: HTML markup is below. Please do not edit.]

Reviewers' comments:

Reviewer's Responses to Questions*

***Comments to the Author*** *1. Is the manuscript technically sound, and do the data support the conclusions?

The manuscript must describe a technically sound piece of scientific research with data that supports the conclusions. Experiments must have been conducted rigorously, with appropriate controls, replication, and sample sizes. The conclusions must be drawn appropriately based on the data presented.

Reviewer #1: Partly

Reviewer #2: Partly*

*2. Has the statistical analysis been performed appropriately and rigorously?

Reviewer #1: N/A

Reviewer #2: Yes*

*3. Have the authors made all data underlying the findings in their manuscript fully available?

The* [*PLOS Data policy*](http://www.plosone.org/static/policies.action#sharing) *requires authors to make all data underlying the findings described in their manuscript fully available without restriction, with rare exception (please refer to the Data Availability Statement in the manuscript PDF file). The data should be provided as part of the manuscript or its supporting information, or deposited to a public repository. For example, in addition to summary statistics, the data points behind means, medians and variance measures should be available. If there are restrictions on publicly sharing data—e.g. participant privacy or use of data from a third party—those must be specified.

Reviewer #1: No

Reviewer #2: Yes*

*4. Is the manuscript presented in an intelligible fashion and written in standard English?

PLOS ONE does not copyedit accepted manuscripts, so the language in submitted articles must be clear, correct, and unambiguous. Any typographical or grammatical errors should be corrected at revision, so please note any specific errors here.*
*Reviewer #1: Yes

Reviewer #2: Yes*

**Reply to Reviewer #1:**

*This paper includes three studies on the interesting and important question of how professional connections and status affect peer evaluations of creative works. The studies consist of an interesting empirical case, which shows an interaction effect between social ties and status on evaluation outcomes. However, we cannot be sure that this has to do with social status/distance rather than, say, project attributes, so the authors conduct 2 additional lab studies on Amazon Turk, enabling to overcome questions of confounding. The find an interaction effect between expertise and social ties, and then show that it occurs only when evaluation is “public.” I mention my concerns below and provide an overall summary at the bottom.*

**We very much appreciate the time and thought you have clearly put into evaluating our manuscript. Your comments have highlighted several areas where we could improve our paper. Below, we explain how we have responded to them. For the sake of convenience, we present your comments (in italics) first and then our response.**

*The first study builds on a dataset the authors have analyzed in 2 previous publications:
Aadland E, Cattani G. Ferriani S. Friends, Gifts and Cliques: Social Proximity and Recognition in Peer-Based Tournament Rituals. Academy of Management Journal. 2018.* [*https://doi.org/10.5465/amj.2016.0437*](https://doi.org/10.5465/amj.2016.0437)*. Aadland E, Cattani G. Ferriani S. The Social Structure of Consecration in Cultural Fields: The Influence of Status and Social Distance in Audience–Candidate Evaluative Processes. Research in the Sociology of Organizations. 2018; 55:129–57. doi: 10.1108/S0733-558X20180000055006

I read the AMJ article, which focused on the role of social distance. The second I admit I have not read in full, but, judging by its abstract, it sounds quite similar to what the authors report here in Study 1. So in a future version of this paper I’d like to include some justification for why Study 1 differs from that second reference. And if it materially doesn’t, I’d recommend for it to be described only briefly in the current paper, saving a lot of space.*

**Thank you for highlighting the need to specify how Study 1 is distinct from the study published in Research in the Sociology of Organizations (RSO). Study 1 differs from the RSO publication because it focuses on and seeks to unpack the interaction between the status of the project members (now labeled *Project Status*) and the social ties between project members and jury members (both direct ties and geodesic distance). In addition, in Study 1 we now include new control variables to assess the nature of the interaction more precisely, as well as rule out alternative explanations for the study’s main findings. In particular, we created controls for the status of the jury members, the experience of the jury members, and a control for jury-project reciprocity. By contrast, the RSO publication focuses only on the main effects of project member status and project-jury social proximity (geodesic distance) – not their interaction – and nor does it include the new controls used in this study.**

**As we explain on page 31 of our manuscript, “understanding how audience evaluations may change with the degree of scrutiny to which they are amenable seems crucial in light of ever-increasing calls for transparency in public life. In this respect, probing the interaction between candidates’ status and audience-candidate connectivity is especially important if only one considers that social proximity between producers and audience members is a constitutive feature of peer-based evaluative settings.”**

*I generally think the studies are well done but outline below my questions/concerns regarding how well they relate to each other, internal validity, and some smaller concerns.*

*Study 1
Measure of status. The authors measure social ties between individuals in the Silver Tag population by whether they previously collaborated. Status is measured using this very same network, the more central one is in the network (the more collaborators), the higher the centrality. That’s certainly not ideal, to measure ties and status using the exact same data, and surely enough the two variables correlate at +0.5. But my bigger concern is the following: for high status (high centrality) individuals to have direct ties with a jury, that means the jury is also high status. That leads to possible alternative interpretations. For example, perhaps it’s not that juries prefer high status projects, it’s that high status juries are more lenient. In practice what this means is that the same care the authors put into measuring attributes of the project, i.e., its sophistication, they should put into measuring attributes of the jury.*

**Thank you for highlighting this very important point. We agree that the care we put** **into measuring attributes of the project should also be put into measuring attributes of the jury. Accordingly, we generated two new control variables that measure attributes of the jury. First, we created the variable *Jury Status* that measures the number of high status jury members over the total number of jury members. Second, we created the variable *Jury Experience* that measures the median experience of the jury members. Both variables turned out to be insignificant in our models.**

*Interpretation of interaction effect: Pg 14: “Specifically, while jury members favour projects created by high-status professionals if they are not connected to them, they favour projects created by low-status professionals to whom they are connected.” The latter part of this sentence may not necessarily be the case. The coefficient on social_ties in outcome ~ B1*social_ties + B2*status + B3*social_ties*status + other¬_terms is: B1 + B3*status. Plugging in the estimated coefficients for B1 and B3 in Table 1, Model 7, we get: 0.489 – 0.466*status. So for status ≈1 (high status), this coefficient is apprx. 0, meaning that for high status actors, social ties play (apprx.) no role. For low status actors (status ≈0), the more social ties the better. To turn the attention on status, the coefficient is: B2 + B3*social_ties ≈1.030 – 0.466*social_ties. So for social status to be have a negative association with outcomes we need 1.030 – 0.466*social_ties < 0. So maybe when social_ties is very high (>=3), status starts having a negative association, but there’s probably not quite enough n in the study to make such a fine-grained claim. But the overall point is that for low to moderate values of social_ties, it’s still good to be high status. After writing all the above, I realized the paper has a further discussion of the results!, which seem to be anticipating what wrote and, as far as I can tell, do not support that original sentence I took issue with on Pg. 14.*

**We commend you for bringing this inconsistency in our discussion of the results to our attention. You are exactly right: as Figures 1 and 2 (reported below for your convenience) indicate, when the value of social ties is higher than 2.21 the effect of status becomes negative. This value is indeed quite high but not so high that no projects exhibit such a value in our dataset (the maximum observed number of jury-project ties is 5). Likewise, Figure 1 shows that the marginal effect of social proximity disappears when status approaches the value 1 (but this is a theoretical value since no jury in our dataset had 100% of its members with a value of Bonacich beta-centrality above the median). In light of the above, we removed the sentence “Specifically, while jury members favour projects created by high-status professionals if they are not connected to them, they favour projects created by low-status professionals to whom they are connected” (p. 14) because the fine-grained discussion of the result does not support this sentence – which you rightly took issue with. We also reconsidered our arguments to reflect more accurately the marginally decreasing effect of status at increasing levels of social proximity.**

**Figure 1. Average Marginal Effects of *Social ties***

**Figure 2. Average Marginal Effects of *Project status***

*The authors should be applauded for pursuing the suggestive observational data with experiments. Here are my concerns with those:*

*Study 2
What is the treatment? It’s priming status or social ties, not randomly manipulating them in reality. What such a treatment really means, I’m not sure, and I’m afraid it’s rather difficult to know. If an experimenter told me “evaluate this video, and pretend you know the people behind it,” I would probably be confused by that additional information and become suspicious of the study’s motivations.” All that said, I think it would be good to include the authors’ discussion or arguments for why priming these measures should be sufficient somewhere in the paper.*

**Thank you for appreciating our effort to combine field data with experimental one. We concur with your point and we are aware that replicating the evaluating setting of our field study would be ideal but is also very hard in online experiments. We tried to do our best and used our scenario-based manipulations as form of contextual priming, in which primed knowledge works as an anchor – i.e., as a standard of comparison for (re) evaluating the target, possibly resulting in contrasting the target away from the prime (Strack & Mussweiler, 1997). In doing so, we relied on prior similar experimental studies that used descriptive texts to prime status (e.g., Sgourev and Althuizen, 2017) or social ties (e.g., Zhao and Xie, 2011). We included these considerations in footnote 3.**

*What is status? In study 1, status was network centrality. That seems like a reasonable measure, as status in sociology tends to entail deference and structural position, and to be conceptually distinct from direct quality or expertise. However, in studies 2 and 3 status is now operationalized as expertise, i.e. “well-known experts” vs “non-experts.” This is a very substantial conceptual change, because now we are talking directly about expertise, and that is what is being manipulated. If the authors really want to equate position in collaboration networks with expertise, and if they are the same why both are not the same as quality, it would be important to include such arguments somewhere early or middle paper.*

**In Study 2 and Study 3, we operationalized status as “well-known experts” because prior sociological research in the creative industry often does not conceptually discern between status and expertise as both are considered a signaling strategy (e.g., Jones, 2002). For instance, in the film industry, Elsbach and Kramer (2003) argue that when catchers were “aware of their greater expertise and ability during a pitch, they were likely to categorize their relationship with the pitcher as one between a high-status expert and a low-status incompetent” (p. 294). Thus, this suggests that *status* and *expertise* are not necessarily considered conceptually distinct in the creative industry.**

**Furthermore, in our status manipulation we qualified ‘experts’ as ‘well-known’ to assign prestige to the commercial’s creators. Indeed, our manipulation checks for status indicated that these commercial’s creators were perceived to be more prestigious advertisers than advertisers described as ‘non-experts.’**

**Finally, to further address your concern, we decided to replicate Study 2 with a different manipulation of status in which we used ‘famous’ and ‘not very famous’ to describe the commercial’s creators. Specifically, we mentioned in the status condition that ‘the authors of the commercial are famous professionals in advertising;’ while, in the no-status condition we specified that ‘the authors of the commercial are not very famous professionals in advertising.’ Our manipulation check revealed the effectiveness of this status manipulation; also, our results replicated the findings obtained in Study 2.** **Given the prior literature pointing to the importance of social ties in shaping a producer’s fame (Lang & Lang 1988, Accominotti 2009), this approach should temper your preoccupations with the potential disconnect in status operationalization criteria between Study 1 (centrality) and Study 2 and 3 (expertise).**

**Please, refer to the Appendix for a complete description of this experiment.**

**Accominotti, F. 2009. Creativity from interaction: Artistic movements and the creativity careers of modern painters. Poetics, 37(3), 267-294.**

**Jones, Candace. 2002. Signaling expertise: How signals shape careers in creative industries. Career creativity: Explorations in the remaking of work, 209-228.**

**Elsbach, Kimberly D., and Roderick M. Kramer. 2003. Assessing creativity in Hollywood pitch meetings: Evidence for a dual-process model of creativity judgments. Academy of Management journal, 46(3): 283-301.**

**Lang, Gladys Engel, and Kurt Lang. 1988. "Recognition and Renown: The Survival of Artistic Reputation." American Journal of Sociology 94 (1): 79-109.**

*Results
It is surprising that neither expertise nor direct ties have a direct effect. If so, this would be among the first studies ever to find that high expertise are not beneficial. This makes me think again about the worry I expressed earlier – what did the treatment actually prime?*

**We appreciate your thoughtful warning. We wish to emphasize, however, that our experiment is a two-way factorial design and the interpretation of the main effects depends on the significance of the interaction. Especially with disordinal interactions our inclination, in keeping with Cohen, is to stay agnostic on the causal meaning of the main effects. As Cohen (2013) suggests: “The significance of the interaction does have implications for interpreting the main effects, which is why it makes sense to test the F for interaction first. If the interaction is not significant, you can test the main effects and interpret them as two separate one-way ANOVAs. But if the interaction is significant, you must be cautious in interpreting the significance of the main effects. It is quite possible that the main effects are misleading” (p. 463). Moreover, if “the interaction is markedly disordinal, it will usually be pointless to perform comparisons on row or column means even if one or both of the main effects are significant. It is more likely that comparisons would focus ultimately on cell means…” (*ibidem*, p. 477). Indeed, as Cohen (2013) further points out, a disordinal interaction as the one in our case “can completely obliterate the main effects” (p. 461).**

**Cohen, B. H. (2013). *Explaining psychological statistics (Fourth edition)*. John Wiley & Sons.**

**Keppel G, Wickens TD. 2004. Design and Analysis: A Researcher’s Handbook, Upper Saddle River, NJ: Pearson.**

*Excluding outliers:
The dependent variable is on a 1 to 7-point scale. The authors say the excluded all observations with > 2.5SD of the mean. Since the DV has no unreasonable values by design, what does it mean to exclude values? Did the authors drop all observations with scores of 1 or 7? What is the justification for this?*

**We removed outliers at 2.5SD in our three experiments to increase the power by reducing the error variance. This is the same reason why we removed inattentive participants by using the two attention checks and the commercial watching time. As Meyvis and van Osselaer (2017, p. 1161) argue, the removal of participants “is often both legitimate and preferable…as this may produce both a more powerful and a more accurate test of the hypothesis.” Also, in order to reduce the researcher’s degrees of freedom, we applied the same criteria throughout our three experiments. Please, see also footnote 5 on p. 21 on this particular point.**

**Finally, with respect to the previous version of the paper, we decided to apply more restrictive criteria to screen out participants and remove those who missed only one correct answer (i.e., the attention check on the commercial and/or the instructional manipulation check; please, see p. 18 for more details). We decided to change the ‘participant exclusion’ strategy because one reviewer rightly pointed out that using the commercial watching time to remove inattentive responses does not ensure that participants really watched the commercial. Since we already had this question about the commercial in the original survey (“What is the commercial about?” footnote # 1 on p. 18), we did not re-run Study 2 but used instead this question to remove participants who answered it incorrectly. Our main results were confirmed. To ensure consistency, in the new two experiments reported in this revised version of our manuscript, we asked the same question about the commercial and removed the wrong responses.**

**Meyvis T., Van Osselaer SMJ. (2017). Increasing the Power of Your Study by Increasing the Effect Size. Journal of Consumer Research, 44(5): 1157-1173.**

*Small issues
Ethnicity: it’s a categorical variable but as far as I can tell the authors treated it as a continuous one, going from 1 to 7. I’d either enter ethnicity into analysis as dummy variables or do 0=white, 1=nonwhite. I’m also puzzled why the authors are control for this, since the experiment had random assignment. To increase power? Would be nice to have the uncontrolled results too.*

**You are correct, we used ethnicity to increase the power. Since you were doubtful about our measure, we now report the analyses without this covariate.**

*Would be nice to have error bars on the observations in the Figures.*

**We added error bars to all the figures representing the bar charts.** *Study 3
Similar concerns to those expressed for Study 2:
This is really about expertise rather than status
Outliers removed*

**Please, refer to our previous response to your similar points regarding Study 2.**

*Results
This study finds priming expertise to have an overall negative (!!!) effect on evaluations, which is very surprising and makes me wonder about the treatment. The results show that no-status projects receive significantly better scores when judged in public, which as the authors point out suggests interesting reputational concerns. However, in the private condition, no-status projects also do better, just not p < 0.05. This latter contrast showing no effect is important for the reputational-concern argument, so it would be helpful to report the following:
What is the power of that test? We have ~50 subjects per cell, after all.*

*Results without removing outliers
It seems the argument is really about the difference (no-status-private – status-private) being smaller, or much smaller, than the difference (no-status-public – status-public). Whether that first difference is in fact nil is not as important. So it would be good to report this “diff-of-diffs” test.*

**We recognize that our Study 3 had a small sample size. To address your concerns, we re-ran Study 3 after recruiting a much larger sample: the analyses now include 397 participants. Please, see pp. 26-28 for the results.**

**As you can now see on Figure 5 (p. 44), which presents more reliable results given the larger sample size of Study 3, the ‘no-status-private – status-private’ difference is almost null and the two mean values are nearly overlapping (**M_status and non-public_ = 4.42, M_no status and non-public_ = 4.44). **On the contrary, the ‘no-status-public – status-public’ difference is significant and the mean values are farther apart** (M_status and public_ = 4.25, M_no status and public_ = 4.58). **Thus, we believe that it should now be sufficiently clear that the first difference is smaller than the second difference even without conducting a “diff-of-diffs” test.**

*Small issues
For the two t-tests with the contrasts, the degrees of freedom df=204 seem off. If it’s ~50 people per cell, comparing two cells should have df ~= 50 + 50 – 2 = 98.*

**The reason for df=204 is that we used the error term from the entire design. This procedure is suggested to run contrast analysis in the between-subject design, when the homogeneity of variance is not violated. To test for this assumption, we used Levene’s test, which was not significant - thus suggesting that the homogeneity of variance was not violated. In our new Study 3, we now have df equals to 393 for the planned contrasts (please, see p. 28): as before, we use the error variance based on the entire sample.**

*Small overall issues
Pg 13: “We clustered the projects on contest month…”
I assume the authors mean clustered standard errors on contest month? Or you mean you included a fixed effect for the contest?
Pg. 14: “We also clustered project on firm…” Same question as above.*

**You are right. We clustered standard errors on contest/month and on firm. We now make it clear in the manuscript. Thanks.**

*Summary
Overall, I thought the authors are after a very interesting question and did a thorough job analyzing the observational data, and then pursuing open questions with experiments. (Although I know interestingness/novelty are not PLOS’s evaluation criteria, if they were I think the paper would score quite high on both.) So my main questions for the authors are
Do we need to include Study 1 here? Are its conclusions not materially reported in the Research in Soc. of Orgs publication? If there are indeed novel aspects to report here, then I would also like to see controls for jury attributes, particularly its status.*

**You reiterate an important question here. As explained before, the main contribution of Study 1 is to uncover and examine the nature of the interaction effect between the status of project members and the social ties connecting project and jury members, while also controlling for additional jury attributes. In particular, we now employ controls for the status of jury members and the experience of jury members, as well as a control for project-jury reciprocity. The RSO publication does not report any material results on interaction effects, nor any of these controls. Thus, the new model specifications build on the results of the RSO publication but also extend them in a way that, we believe, is theoretically novel and interesting.**

*Studies 2 and 3 are about expertise, and so their connection with Study 1’s focus on status is not yet convincing. Can you provide this to the reader?
Studies 2 and 3 have peculiar direct effects, raising concerns about what did the treatment really do?
Would like to see Studies 2 and 3 analyzed without removing score-outliers, or at least justifying why this is done.*

**Please, refer to our previous responses regarding these points.**

*Thank you for the opportunity to engage with your interesting work!*

**Again, we would like to thank you for your very thoughtful and constructive comments.**

**Reply to Reviewer #2**

*I like to believe that the study has merit – especially in terms of its execution –, but in its current form it is quite convoluted in some parts and lacks essential details in others. The former can be fixed; the latter is harder to evaluate since the authors do not put the reader in the situation of being able to judge important aspects of the studies with sufficient detail. I will attempt to provide suggestions.*

**We would like to thank you for pushing us further to improve our paper. We have done our best and think the paper has improved: we hope that you agree with us. Below, we explain how we have responded to your comments. For the sake of convenience, we present your comments (in italics) first and then our response.**

*Major points:
I had a hard time assessing the real merit of this study. Not because I am worried that others have done a lot of this work already, but because I kept wondering whether the authors themselves have already done and published much of the work presented here. Study 1 in particular constantly refers to and directly quote from work that (a subset of) the authors have published elsewhere (pg. 9ff). It appears that authors borrow both data and methodology from previous papers that the authors published, so that it's difficult to evaluate not only what exactly is novel here, but also to what extent the same analyses and results are not being published multiple times. Making this clearer is of utmost importance from a scientific point of view.*

**Thank you for highlighting the need to specify the novelty of this work relative to our previously published papers. As you rightly point out and as we very openly explain in the manuscript Study 1 draws on previously published data and methods. However, it differentiates itself significantly from those studies by focusing on and unpacking the interaction between the status of the project members (now labeled *Project Status*) and the social ties between project members and jury members (both direct ties and geodesic distance). None of the previously published studies investigates or theorizes on the interaction between status and social ties. Nor any of those studies elaborates on how evaluative outcomes may change depending on the degree of scrutiny to which audience members are amenable – an especially important point that seems crucial in light of increasing calls for transparency in public life. Furthermore, Study 1 now incorporates novel control variables not reported in the previously published papers to assess the nature of the interaction more precisely. In particular, we introduce controls for the status of the jury members and the experience of the jury members. These new model specifications clearly build on the results of the RSO publication but also extend them in a way that, we believe, is theoretically novel and interesting. Study 2 and 3 are both new from a conceptual, empirical and analytical perspective. In short, this manuscript advances theoretical arguments that we had not developed in our previous research, but also adopts an original analytical approach that builds significantly on our published work while departing from it in several important ways.**

*More generally, I found Study 1 to be most problematic based on the information (not) provided in the text. Study 2 and 3 have an interesting angle but are not incentive-compatible. Participants were simply paid a show-up fee, but the elicitation protocol of their subsequent decisions is crude and it’s not clear why we should treat the stated preferences as truthful representations. I am an experimental economist and I understand that the authors have a different background, but these are the issues we are concerned with. Hence, I would encourage the authors to engage in a more convincing dialogue with the reader. Below, a selection of challenges that I had with the studies.*

**Thank you for bringing these challenges to our attention. We have done our best to address them. Below, we elaborate on our approach.**

*Study 1:
- Do I understand correctly that all of this discussion is based on a sample of 9 observations? The authors never clearly state it, but the referenced Table 1 gives that impression. Obviously, this is highly problematic in and of itself. But what’s more, the authors omit important information regarding the exact procedure of the data collection (e.g., who did the interviews, duration, compensation etc.). It’s not ok to simply refer to another publication for these details – the reader needs to understand this from reading this paper.*

**We apologize for the lack of clarity and information in our presentation of the insights drawn from the interviews we conducted with a panel of field insiders. We now provide more information on the exact procedures of the data collection (how we approached the setting and obtained access, the number of interviews, informed consent, topics covered, interviewer identity, duration, compensation, etc.) on pp. 8-10. We conducted 19 interviews in total (please, see Table 1 on p. 37).**

*- Independent variable: the paper refers to concepts without defining them and assumes that the reader knows them already. Don’t. Exemplary, the definition of the independent variable is confusing. Rather than referring the reader to another paper that summarizes the methodology, it would be good to have an informed discussion right here to point out pros and cons and why exactly this measure delivers what the authors are after. After all, these variables are central to the investigation and the reader needs to be convinced of its validity.*

**Thank you for asking us to explain our measure of status in more detail. We now illustrate how this measure of beta-centrality capture status on p. 12 and offer further details in footnote 1. In particular, we illustrate how this eigenvector centrality measure captures a focal advertising professional’s position of importance in the network as a function of the number and centrality of the advertising peers connected to the focal professional. The status of these peers is in turn a function of the number and centrality of the professional peers connected to them, and so on. Consequently, the beta-centrality scores assign each professional to their place in a hierarchy of positions of importance in the global network. In general, this measure can be interpreted as both a measure of degree centrality or status; the difference is given by the parameter β. When β approaches zero the beta-centrality yields values close to degree centrality. When β > 0 the formula takes into account other nodes centrality. Greater values of b assign higher weights to more central nodes. The weighting factor β in our case was set equal to the reciprocal of the largest eigenvalue of the relational matrix (this is because the reciprocal of the largest eigenvalue represents the upper bound), in this way, the higher the status of one professional, the more s/he is enhancing the status of another professional in the network. This is a commonly used approach to derive status ordering from relational data (Podolny, 1993, 1994; Benjamin & Podolny, 1999; Podolny, 2001).**

*I am also worried about the validity of the variable ‘social ties’, as this seems to be a quite crude measure. What about working at the same institution / department etc., all of which could be measured? One can have close social ties without having worked on a previous project. The authors should attempt to be more convincing here.*

**We employed several measures in Study 1 to capture different facets of ties between project members and jury members. In addition to the *Social Ties* measure that captures direct ties, we further explored the influence of indirect ties using the measure *Social Proximity*. When generating this measure, we first calculated the median geodesic distance between each project member and the jury members. In accordance with the six degrees of separation theory, we then grouped together individual producers with a degree of separation from jurors equal to or greater than 6 and assigned them the value 6. To facilitate the interpretation of the results, we measured the variable in terms of nearness between jury members and producers. We measured nearness by computing the reciprocal of the median geodesic distance between each project member and the jury members. As our unit of analysis is the project, we created the social proximity variable by taking the median of each project member’s median distance from jury members.**

**We also captured project members and jury members working on the same project, and/or working in the same firm, through our dummy variable *Conflict of Interest*. In the event of a conflict of interest, the juror in question cannot take part in the evaluation of the project in question.**

**We captured whether project and jury members collaborated in the past and won an award with the dummy variable *Prior Positive Co-experience*.**

**We controlled for potential project-jury reciprocity dynamics, as some project members may have served as jurors in the past and in this role rewarded current jury members.**

**We hope that all these additional variables get closer to capturing various facets of social proximity.**

**Finally, we added a new paragraph in the conclusions where we acknowledge the possibility that other types of social ties – not captured in our data – may cut across the audience-candidate interface, and discuss this as a potential avenue for future inquiry.**

*Study 2:
- The authors talk a lot about the need to exclude participants, but it remains vague at times. For example: “Since we required participants to watch a commercial that lasted 55 seconds, we removed those participants who did not watch and/or spent too much time watching the video.” How did you ensure that participants really watched the video? By recording whether they clicked play? Clicking and consuming are two different things and it’s not unlikely that this introduced differential exposure to the message that is correlated with unobserved individual characteristics.*

**You raised a very important question here. Following your advice, we decided to apply more restrictive criteria to screen out participants and remove those who missed only one correct answer (i.e., the attention check on the commercial and/or the instructional manipulation check; please, see p. 18 for more details). We decided to change the ‘participant exclusion’ strategy because we concur with you that using the commercial watching time to remove inattentive responses does not ensure that participants really watched the commercial. Since we already had this question about the commercial in the original survey (“What is the commercial about?” footnote # 1 on p. 18), we did not re-run Study 2 but used instead this question to remove participants who answered it incorrectly. Our main results were confirmed. To ensure consistency, in the new two experiments reported in this revised version of our manuscript, we asked the same question about the commercial and removed the wrong responses.**

*Study 3:
- Why is both the sample size and the incentivization (show-up fee) so different from study 2? Given that you motivate study 3 to dig deeper into what’s going on in study 2, you want to avoid any additional noise by keeping as much of the setting constant as possible. In addition, at no point do you motivate the reason for collecting the amount of data. Is this informed by some sort of power calculation, expected effect sizes etc.? Convince the reader that the presented results are statistically reliable. This, in turn, could then also explain why your ‘award propensity’ results are not how you expected them to be. The citations that you use there to justify them are only convincing if you had sufficient power to begin with.*

**We recognize that our Study 3 had a small sample size. To address your main concerns, we re-ran Study 3 by recruiting a larger sample: our analyses now include 397 participants. Please, see pp. 26-28 for the results.**

**We also replicated the results of Study 2 by using another scenario for the evaluating setting and a different manipulation of status. Please, see the Appendix for more details.**

**We did our best to improve our experiments and we hope our results are now more convincing with the two new studies. We decided to simply pay participants a show-up fee in the new experiments in order be consistent with Study 2.**

*Minor points:
- Abstract could be streamlined and state the results clearer*

**We did so.**

*- Intro takes too long to get to its main contribution and much of the cited literature could be better placed in a separate literature section. Overall, the intro is quite lengthy, and it takes the authors too long to get to their own contribution and goal of this paper*

**We streamlined the introduction significantly (it is now approximately one page shorter).**

*- pg. 4: the authors miss relevant literature especially from the experimental economics and social psychology literature (e.g., Gino & Moore, 2007; Charness et al., 2007; Dimant, 2019; Hugh-Jones & Ooi, 2017 – and the papers cited in these papers), so that the authors overstate their contribution at times. I suggest engaging with this literature in more detail.*

**Thank you! We incorporated most of the authors you suggested. We found especially relevant Dimant (2019), Charness et al. (2007), and Hugh-Jones & Ooi (2017).**

*Bibliography
Gino, F., & Moore, D. A. (2007). Effects of task difficulty on use of advice. Journal of Behavioral Decision Making, 20(1), 21-35.
Charness, G., Rigotti, L., & Rustichini, A. (2007). Individual behavior and group membership. American Economic Review, 97(4), 1340-1352.
Dimant, E. (2019). Contagion of pro-and anti-social behavior among peers and the role of social proximity. Journal of Economic Psychology, 73, 66-88.
Hugh-Jones, D., & Ooi, J. (2017). Where do fairness preferences come from? Norm transmission in a teen friendship network (No. 2017-02). School of Economics, University of East Anglia, Norwich, UK.*

**Again, we would like to thank you for your thoughtful and constructive comments.**

6. PLOS authors have the option to publish the peer review history of their article. If published, this will include your full peer review and any attached files.


If you choose “no”, your identity will remain anonymous but your review may still be made public.


**Do you want your identity to be public for this peer review?** For information about this choice, including consent withdrawal, please see our [Privacy Policy](https://www.plos.org/privacy-policy).

Reviewer #1: No

Reviewer #2: No


[NOTE: If reviewer comments were submitted as an attachment file, they will be attached to this email and accessible via the submission site. Please log into your account, locate the manuscript record, and check for the action link "View Attachments". If this link does not appear, there are no attachment files to be viewed.]


While revising your submission, please upload your figure files to the Preflight Analysis and Conversion Engine (PACE) digital diagnostic tool, <https://pacev2.apexcovantage.com/>. PACE helps ensure that figures meet PLOS requirements. To use PACE, you must first register as a user. Registration is free. Then, login and navigate to the UPLOAD tab, where you will find detailed instructions on how to use the tool. If you encounter any issues or have any questions when using PACE, please email us at [figures@plos.org](mailto:figures@plos.org). Please note that Supporting Information files do not need this step.
