# Supplementary material for: Reflecting glory or deflecting stigma? The interplay between status and social proximity in peer evaluations
Source: PLoS One. 2020 Sep 25;15(9):e0238651. doi: 10.1371/journal.pone.0238651 (PMC7518619; doi:10.1371/journal.pone.0238651)
Supplement: Attachment — Submitted filename: Report_Cattani_PLOS_ONE_2020.docx [file pone.0238651.s007.docx]

**Report for „ The Interplay Between Status and Social Proximity in Peer Evaluation: A Socio-**

**Cognitive Perspective on Reward Allocation “**

Manuscript ID: PONE-D-20-09850

SUMMARY

The authors test the role of social status and social proximity for evaluation and recognition choices such as the allocations of awards with field data and two experiments. Based on the literature, they ask if status of the candidate and social proximity between candidate and jury members act as substitutes or complements in how they combine to produce evaluative outcomes. Based on observational data from an award in advertising industry in Norway and two experiments, they show a negative interaction between status and social proximity. The experiment suggests that when evaluations are public, social proximity tampers the effect of status on recognition, but not when decisions are private. Thus, reputational concerns of the jury members may lead to a substitutional effect in their setting.

RECOMMENDATION TO THE EDITOR:

I recommend a revise and resubmit. I think the topic is very interesting and relevant and the combination of field data and an experiment is interesting and promising. Unfortunately, the design of the experiments has many problems, which make me unsure whether the paper should be accepted for publication without major changes in the design and possibly even a replication of the experimental studies. Especially, I am not convinced by the treatment manipulations and by the interpretation of the behavior they observe, see below. However, I still would like to give the authors a chance to resubmit a modified version and explain their choices.

MAIN COMMENTS

I don’t know this precise field of literature well, so I would leave it to another referee to judge the novelty and originality of the contribution this study makes to the existing literature. If the empirical/experimental evidence is sufficiently novel, I think the topic is very relevant and interesting in many areas and professions. One application certainly is peer review in academia.

STUDY DESIGN:

- It’s a nice feature of the article, that the authors try to establish additional external validity and try to validate their own interpretation of their findings by interviewing professionals in the field.
- FIELD STUDY: Can the authors elaborate a bit more on how project quality is controlled for? It seems like this is not perfectly captured by their variables and it is very likely to be correlated to project status. This might be a problem for the identification in the field setting.
- EXPERIMENT 1:
  - I find it a bit problematic that the vignettes only contain information about creators social status and social ties (p.20). This makes the situation highly hypothetical and also can create experimenter demand effects.
  - The social ties condition is highly abstract--“imagine you have collaborated with them in the past”—and does not really induce true social ties. There are many experiments that truly manipulate “social proximity”, i.e. that really induce a social distance or connection but priming them on common preferences, creating groups. It would have been even possible here to actually have subjects work together on a task and then match them for the jury-candidate experiment… There are many good examples in previous studies and I don’t understand why the authors don’t use them. I’m not convinced this manipulation has the desired effect.
  - DV (p.21): Subjects watch one commercial and should then say how likely they are to award it. How should they make this choice? They have nothing to base their choice on, i.e. no other commercials to compare it to. Because of this, the manipulations by the experimenter might actually have a larger effect than they would have otherwise.
- EXPERIMENT 2:
  - The dampened signaling effect of status if a jury member and a candidate have close social ties does NOT work in their design because they HAVE NO actual social ties. This mechanism cannot be detected in their experiment (p.24). This also shows that their manipulation of social proximity is highly problematic for their purpose (see above).

ADDITIONAL MINOR COMMENTS

- In Experiment 1, the authors mix “status” and “expertise” in their treatment manipulation which I find very questionable. What is the reason for doing this? It seems easy to disentangle the two and therefore to avoid this confound. Giving an “expert cue” and interpreting its effect as “status” seems like an interpretation that is probably not warranted.
- Especially without pre-registration, removing any subjects from the analysis is questionable, e.g. removing the outliers, p.22.
- Overall, I recommend to shorten and streamline the text.
- Tables in the text instead of in the appendix would have helped a lot to make the paper more readable.
