# Supplementary material for: Reflecting glory or deflecting stigma? The interplay between status and social proximity in peer evaluations
Source: PLoS One. 2020 Sep 25;15(9):e0238651. doi: 10.1371/journal.pone.0238651 (PMC7518619; doi:10.1371/journal.pone.0238651)
Supplement: Attachment — Submitted filename: Response to Reviewers.docx [file pone.0238651.s009.docx]

**Reply to Editor**

We appreciate the time and thought you have put into your evaluation of our manuscript. Since the first two reviewers accepted the paper, we only addressed the comments of reviewer 3.

We hope you will find this revised version of our manuscript to deserve publication in *PLOS ONE*. Again, thanks for your guidance throughout the review process. We remain at your disposal for any further clarification.

**Reviewer #1: Thanks for accepting the paper.**

**Reviewer #2: Thanks for accepting the paper.**

**Reply to Reviewer #3**: **Thanks for your comments. Below, we respond to them in bold.**

The authors re-write part of the paper and provided additional evidence to address some of the reviewers’ concerns. Overall, I appreciate that the authors provide new evidence and modified some parts in the paper, however, the changes do not fully address some major concerns I had (have) with the paper. Therefore, I was on the fence whether to reject or accept the paper before and the revised version has not convinced me to change my mind in either direction.

**Again, we very much appreciate the time and thought you have put into re-evaluating our manuscript.**

Study 5

Study 5 replicates part of study 2 by varying status when social ties are present and evaluations are public. By modifying the way close social ties are depicted in the experiment, the authors provide a robustness effect of the finding that with close social ties, higher status negatively affects the likelihood to give an award. Contrary to study 2, social ties are not varied and the interaction effect is not tested. While providing a robustness check is valuable per se, I have two questions: Which exact concern does this design variant address? Why is the interaction not tested, i.e. why is only part of the result from study 2 replicated? I suggest to either address these two questions and explain the value added or to just leave out this replication in the final paper.

**We designed Study 5 to focus on the case with social ties because we thought this case was theoretically more interesting. This was also in line with Study 3, where we kept social ties constant to disentangle the mechanism underlying the negative effect of status. Furthermore, we had already showed the existence of the interaction between status and social ties in Study 1 (Field Study), Study 2 (experiment) and Study 4 (experiment). Following your suggestion, we decided to remove Study 5 from the revised version of our manuscript: this study was indeed only a robustness check and it is no longer necessary in the final version.**

Study 3

The authors suggest that two mechanisms can drive an interaction of status and social ties: information and social pressure. Study 3 varies whether evaluations are public or not. Social ties are kept constant (close ties) and status is varied as before.

p. 28: “…we could rule out the alternative explanation that status and social (ties) information operate as substitutive information devices” As also pointed out in my first round review, I think the authors have to be more careful with their conclusion here. (1) I agree that it’s probably unlikely to play a major role in your experiment due to the other previous findings, but the conclusion is too strong here, it cannot be “ruled out”. (2) information is a mechanism that they cannot test with the current design since there is no information that comes with close social ties in the way they are modelled in the experiment (purely based on descriptions in the vignettes). Thus, this channel seems to be shut down in this study, i.e. they don’t test it (which is okay, just don’t state otherwise).

**You make a very important point. Following your suggestion, we toned down our conclusion.**

**We hope we have addressed all of your comments. Again, thanks for your very thoughtful and constructive comments.**
